# Influence of Internet Celebrity Medical Experts on COVID-19 Vaccination Intention of Young Adults: An Empirical Study From China

**DOI:** 10.3389/fpubh.2022.887913

**Published:** 2022-04-12

**Authors:** Jianliang Wei, Minjun Zhao, Fei Meng, Jingjing Chen, Yingying Xu

**Affiliations:** ^1^School of Management Science and Engineering (MSE), Zhejiang Gongshang University, Hangzhou, China; ^2^School of MSE, Zhejiang Gongshang University, Hangzhou, China; ^3^Zhejiang Police College, Hangzhou, China; ^4^Zhijiang College, Zhejiang University of Technology, Hangzhou, China; ^5^School of Economics and Management, University of Science and Technology Beijing, Beijing, China

**Keywords:** medical expert, internet celebrity, COVID-19, vaccination, influence factor

## Abstract

With the continuous expansion of COVID-19, many medical experts with the characteristics of “Internet Celebrities” are increasingly influencing people's vaccination behavior, which is crucial for overall social welfare. To explore the influence of Internet celebrity medical experts on people's vaccination against COVID-19, this study constructed a conceptual model of COVID-19 vaccination intention based on the professionalism, morality, interaction dimension, and information content of Internet celebrity medical experts, to generate perceived value by establishing a trusting relationship between them and the influenced people. The empirical analysis shows that interactivity and information content are important factors determining the influence of Internet celebrity medical experts. In the context of high demands for COVID-19 vaccines, it is more effective to influence vaccination intention through strong demand than through generating trust. The empirical analysis shows that Internet celebrity medical experts have a significant role in COVID-19 vaccination, and interactivity and information content are two important factors determining the influence. Through the connection of information-demand, Internet celebrity medical experts can greatly influence the perceived value, by coaction with trust to influence the final intention. Therefore, the COVID-19 vaccination persuasion information released by Internet celebrity medical experts should be elaborately organized and demonstrated, especially from the demand aspect, and government could put more resources to support the information to spread.

## Introduction

With the growing popularity of social platforms, social networks have increasingly become an important source of information for users. A survey has found that nearly 70% of users take the Internet as their main information source (1). Health information has also become an important type of online information, especially with the outbreak of COVID-19, not only the behavior of users to obtain epidemic information through the Internet has increased significantly, but also people's emotions are easily affected by media reports on COVID-19 and the spread of health information.

The influence of medical experts has always existed in social networks. Some researchers compared the influence of medical experts and non-professional celebrities on credibility and public intention behavior in the network environment and found that participants generally trusted medical experts in comparison (2). Accurate and objective information dissemination is crucial in response to COVID-19, and many elites in the medical community have joined the media coverage of the Novel Coronavirus pandemic. Prevention and control experts use language that is concise and easy to understand and broadcast through the media promptly, by which they create a great impact on the trend of the epidemic, the stability of public opinion, and control the spread of rumors. Among them, some medical doctors disseminated pandemic-related information with close logic and vivid language through diversified communication channels, which became the authoritative information source for public awareness, prevention, and treatment. Medical experts also gradually became the “Internet celebrities” of the whole people, and their voices became powerful enough to send COVID-19 information and change people's behavior. In this article, we define the Internet celebrity medical expert as a medical professional who has high reputation in the COVID-19 field and has a lot of fans on the Internet based on his/her words online.

However, due to the uncertainty and abruptness of the pandemic, as well as the coexistence of scientific and false information on social networks, the information received by users is extensive and fragmented, through which the public create a huge demand for authoritative information when making vaccination decisions, and the phenomenon of “vaccine hesitation” always exists. Many people resist vaccination because of concerns about safety and potential side effects. In the context of repeated outbreaks of COVID-19 and the emergence of new strains, it is of great significance to improve the vaccination coverage rate of the whole society, effectively perform the role of Internet celebrity medical experts, and clarify the key influencing factors, thus providing a theoretical basis for policymaking.

There are three contributions of this article, first is putting out a novel perspective to analyze the COVID-19 vaccination intention, second is giving out a framework to scale the influence of Internet celebrity medical experts, and third, finding information content as the most important factor. The rest of the article is organized as follows: Section Literature Review is the review of relative works of literature. The variables and hypotheses are put forward in Section Concept Model and Hypothesis, and the conceptual model was also constructed. In Section Empirical Analysis and Discussions, data based on the questionnaire were collected and path analysis was conducted, and mainly results are discussed. Conclusions are in the final section.

## Literature Review

### Opinion Leader Perspective

People's attitude toward a certain behavior determines their intention to act, and the attitude itself comes from the belief of the corresponding behavior. Based on this, the functional model Theory of Reasoned Action (TRA) proposed by Fishbein and Ajzen (3) provides an overall framework for the mechanism of information transmission. Consumers will combine their expectation of the probability of realizing a certain function with the perceived value (expectancy–value scheme) to form a unique will (3). However, opinion leaders can amplify the effect of information dissemination. For example, a trusted opinion leader or an opinion leader who is considered an expert (such as Michael Jordan endorses sneakers) will enhance the persuasion of advertising information (4). Professionalism, reliability, affinity, and other characteristics can effectively affect trust. In the field of health information dissemination, some researchers have found that credibility and information participation are important variables affecting the trust process (5). Not only does the professionalism of opinion leaders have a significant impact on their work or interests, but the attractiveness of appearance and quality is also very important (6).

### Vaccination Perspective

Around the development and implementation of vaccines, many studies on vaccination intention have been carried out. As in the study of Abbas et al., factors among adults that promote every year vaccination include older people, perception of vaccine high effectiveness, high income, and no out-of-pocket expenses, and barrier factors include the lack of health insurance, dislike injections, perception of low vaccine efficacy, low awareness of influenza infection risk, and high perception of dangerous side effects (7). As for the role of medical care, Lv's research statistics show that people who receive information from television, community committees, or doctors are more likely to be vaccinated than those who are not (8). Mo and Lau further pointed out directly that the recommendation of healthcare professionals is an important promoter of the influenza vaccine (9).

As COVID-19 vaccines are implemented, COVID-19 vaccination has become the focus of researchers' attention. Wake's review summarizes more than 20 factors related to COVID-19 vaccination intentions (10). Some researchers have suggested that the main factors driving vaccination include age 30–49 years, high level of education, previous influenza vaccination history, trust in vaccine effectiveness, and close attention to the latest vaccine news (11). Hossian et al examined the intention of vaccination on university students using multivariable logistic regression analysis, and find that most of the items of Health Belief Model (HBM) were significantly associated with the positive intention (12). Not only personal perspective, Nery et al. conducted a study from the perspective of organizational support, and discovered the relationship between payment and intention (13).

On the role of professionals, Thaker's study showed that trust in experts and general hesitation about vaccines were significantly associated with COVID-19 vaccination intentions (14). Campaigns by trustworthy scientific experts with information that addresses widespread concerns about vaccines may help increase the use of COVID-19 vaccines. Even party elite can help to increase the vaccination intention, through the way of cues (15). There is also a study which took the opinion leader as a control variable, and found that they are significantly and positively correlated with COVID-19 vaccine uptake intention (16). In the research conducted by Shmueli, he found that opinion leaders on social media expressing support can benefit the vaccination intention (17).

To sum up, currently, there are only a few studies on the intention to vaccinate against COVID-19 from the perspective of Internet celebrity medical experts. However, in China and other practices, there is an order of magnitude difference in the online influence of medical experts such as Zhang Wenhong, who is an Internet celebrity compared to traditional medical experts. It is important to clarify the influence mechanism for Internet celebrities to exert a positive effect on people's intention to vaccinate.

## Concept Model and Hypothesis

Although there are some differences between “Internet celebrity medical expert” and “common Internet celebrity,” for example, Internet celebrity medical experts are top professionals in the field of COVID-19, the number of Internet celebrity medical experts are very few, usually just one or two. And Internet celebrity medical experts generally have a high social position in real society and maybe very important employees in an official institution. But they also share some common features, such as they are Internet celebrities, and a lot of people online are their fans, like to listen to their words, and follow their point of view. Also, they frequently show up in the public online and release attractive information.

### Variable Definition and Research Hypothesis

#### Professionalism

Professional knowledge is the key factor for opinion leaders to effectively change the attitude, will, and behavior of the public, and is the most important dimension affecting credibility. Experts are considered spokesmen with high professional quality, experience, and status, and their words will arouse people's thinking and imitate their behaviors. Such trust and recognition can improve the effectiveness, credibility, and compliance of the information transmitted (18). In its health-based messages, Public Health England (PHE) is increasingly using healthcare experts to speak for campaigns and is emphasizing their professions. Because medical experts are defined as recognized authorities, their information is more reliable than that of ordinary people (19). In a fast-moving and high-tech field like COVID-19 vaccines, expertise is crucial. Based on this, this study proposed the hypothesis:

**H1:** The professionalism of Internet celebrity medical experts can significantly affect people's trust in COVID-19 vaccination.

#### Morality

People tend to seek a balance between morality and behavior, which implies that moral approval is an essential element of the individual. When people perceive the morality of opinion leaders, they will process information through moral sense and identify with opinion leaders to strengthen their moral identity. In this way, opinion leaders shape people's self-awareness. On the other hand, when people feel that their moral norms have been touched, or find that the morality of opinion leaders deviates from their expectations, they may have a negative attitude toward the recommended content. It can be said that moral identity has a greater influence on the process of guiding one's cognition and behavior (20). In summary, and referring to McCracken, the trust of opinion leaders is a broad and multidimensional model (21). We believe that morality is a relatively important aspect, especially in the controversial field of COVID-19 vaccine. Based on this, this study proposed the hypothesis:

**H2:** The morality of Internet celebrity medical experts will significantly affect people's trust in COVID-19 vaccination.

#### Interaction

After being exposed to the information of opinion leaders on the media for a long time, the public will have a sense of identity and closeness to them, and the image characteristics of opinion leaders can gain the public's sense of belonging to them to a certain extent. If opinion leaders actively participate in the interaction, the public can have a deeper sense of identity and thus have more trust in opinion leaders. According to Horton and Richard Wohl's research, this sense of close interaction with opinion leaders can be described as a kind of social relation (22), when people develop a strong sense of social relations, they will often fantasize about the relationship with the opinion leader, reflect a deeper recognition, this identity will lead to the change of attitude and behavior (23). Interaction is one of the key characteristics of Internet celebrity medical experts. When it comes to COVID-19 vaccination, people often have their concerns, and interaction with Internet celebrity medical experts is an important source of trust. Based on this, this study proposed the hypothesis:

**H3:** The interaction of Internet celebrity medical experts can significantly affect people's trust in COVID-19 vaccination.

#### Information Content

Xu and Pratt pointed out that the words released by online influencer plays an important role in meeting people's information seeking needs (24). If opinion leaders can maintain a high-frequency output of certain content quality, they can not only provide users with strong information support but also create a more stable emotional foundation with fans, therefore increasing their trust in opinion leaders. In the context of COVID-19, not only the pandemic itself is evolving dynamically, but also the types, technical routes, and versions of vaccines are constantly updated, making the relationship between content and trust even closer. At the same time, the fashionable and affinitive words which Internet celebrity medical expert used, also can help the public generate trust. Based on this, this study proposed the hypothesis:

**H4:** Information released by Internet celebrity medical experts can significantly affect people's trust in COVID-19 vaccination.

#### Demand Intensity

Demand intensity has always been an important factor affecting perceived value. Vaccine demand has been taken as an important indicator by some researchers in studies on influencing factors of existing COVID-19 vaccines, especially in underdeveloped countries (25). In the context of COVID-19, on one hand, repeated outbreaks make people's demand for vaccination exists invariably. On the other hand, the scarcity or even unavailability of vaccines in some countries and regions contributes to a higher degree of the perceived value of vaccination (26). Based on this, this study proposed the hypothesis:

**H5:** Demand intensity can significantly affect people's perceived value of COVID-19 vaccination.

#### Trust

Trust, through the establishment of an emotional connection between objects, is a critical part for people to generate action intention and decision-making behavior (27). When an object is seen as more trustworthy, the target group has a higher intention to cooperate (28). When trust occurs, it becomes natural to produce behavior and result expectation, and it is easier to believe that behavior decisions can achieve one's expectation. For COVID-19 vaccination, trust is generally considered to be closely related to vaccination intention, including trust in government (29) and vaccine (30). Based on this, this study proposed the hypothesis:

**H6:** Public trust in COVID-19 vaccination can significantly affect vaccination intention.

#### Perceived Value

Perceived value is also a variable often involved in the study of behavioral intention (31). Due to the special function of vaccines, perceived value is one of the most important influencing factors in the studies on influencing factors of vaccination (32). In the context of COVID-19, the effectiveness of vaccines, or to what extent and for how long, has been a topic of specific controversy, and we believe that the perceived value of vaccination is also a critical factor influencing people's intention to vaccinate. Based on this, this study proposed the hypothesis:

**H7:** Perceived value of COVID-19 vaccination can significantly affect vaccination intention.

### Model Construction

Based on the above analysis, this study selected perceived value and trust as double mediating variables based on the special situation of the healthcare field. Meanwhile, it is proposed to use opinion leaders' professionalism, morality, interaction, and information as the observed variables of Trust, and take vaccine demand intensity as the construction dimension of perceived value. Finally, the influence on vaccination intention is realized through users' trust and perceived value of vaccination.

**Figure 1:**
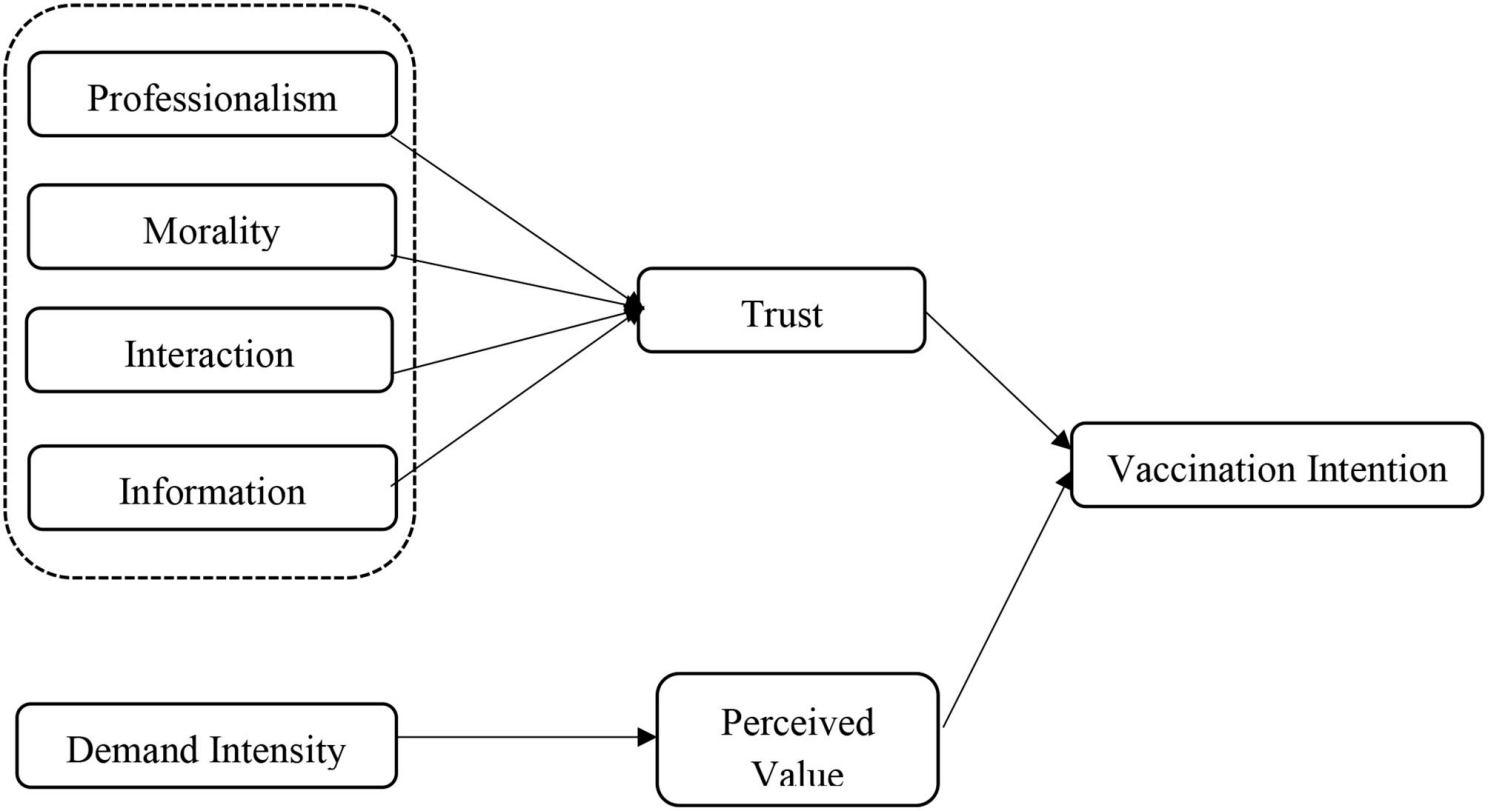
Concept model.

The conceptual model of this study is shown in Figure 1.

## Empirical Analysis and Discussion

### Measurement Variables and Questionnaire Design

Based on the proposed model, we defined specific measurement items by referring to the basis of domestic and foreign research scales, as shown in Table 1.

**Table 1:** Variable measuring scale.

**Variable**	**Item**	**Sources**
Professionalism	• P1. This Internet celebrity medical expert is familiar with the relevant knowledge in the field of COVID-19 vaccine • P2. This Internet celebrity medical expert is very professional in the field of COVID-19 prevention and treatment • P3. This Internet celebrity medical expert has profound knowledge and rich knowledge reserve	Netemeyer and Bearden (33 )
Morality	• M1. This Internet celebrity medical expert treats his work with a dedicated attitude, which will deliver positive energy to the society • M2. The Internet celebrity medical expert has a unique personality (such as warm and height of thought) • M3. This Internet celebrity medical expert is widely recognized by the society	Netemeyer and Bearden (33)
Interaction	• I1. This Internet celebrity medical expert often responds to people's questions • I2. This Internet celebrity medical expert often participates in activities to get closer to the public • I3. If there is an opportunity, I will actively communicate with the Internet celebrity medical expert • I4. The post/information of this Internet celebrity medical expert always gets many replies	Kim and Kim (34)
Information content	• IC1. This online celebrity medical expert publishes COVID-19 related information with high frequency or regularity • IC2. The COVID-19 related information released by this Internet celebrity medical expert is down-to-earth • IC3. The COVID-19 related information released by this Internet celebrity medical expert is very timely and fits hot topics • IC4. This Internet celebrity medical expert will release COVID-19 related information in trendy language or character • IC5. This Internet celebrity medical expert recommends information using interesting pictures • IC6. This Internet celebrity medical expert recommendation information will be made using well-made videos • IC7. The COVID-19 related information released by this Internet celebrity medical expert can basically meet your needs	Kim and Kim (34)
Trust	• T1. I regard the Internet celebrity medical expert as a natural and ordinary person • T2. I think the Internet celebrity medical expert treats us like a friend • T3. If I see reports about him on different media channels/platforms, I will read them carefully • T4. I want to meet him in person • T5. I think the comments of the Internet celebrity medical expert can be trusted completely • T6. The public are very loyal to the Internet celebrity medical expert	Yang and Shim (35)
Demand intensity	• DI1. I think the current situation of COVID-19 is very serious • DI2. I have a serious fear of contracting the Novel Coronavirus • DI3. I think the complications of the Novel Coronavirus are serious • DI4. I think it is very necessary to get vaccinated against COVID-19 now	Bonnevie et al. (36)
Perceived value	• PV1. The government closely monitors the safety of COVID-19 vaccines • PV2. I think the coronavirus vaccine is safe • PV3. Getting vaccinated against COVID-19 is the best way to protect yourself from the virus • PV4. If everyone around me is vaccinated against COVID-19, I will get it too	Bonnevie et al. (36)
Vaccination intention	• VI1. The COVID-19 vaccine recommended by the Internet celebrity medical expert will be my first choice • VI2. I am willing to get the COVID-19 vaccine recommended by the Internet celebrity's medical experts • VI3. I would advise my friends to get vaccinated against COVID-19	Kim and Kim (34)

It mainly covers eight variables including characteristics of medical experts (professionalism, morality, interaction, information content), trust, demand intensity, perceived value, and vaccination intention. The questionnaire of this study consists of two parts: one is the basic information of interviewees, and the other is the measurement of the five variables mentioned above. In the second part of the questionnaire, five-point Likert Scale was used to measure, in which five scales were defined from “disagree” to “strongly agree,” and corresponding scores were assigned from 1 to 5. The degree of agreement increased with the increase of scores.

### Questionnaire Analysis

Therefore, the research objects of this study are mainly young people aged 18–30 years, and questionnaires are distributed online. The number of valid questionnaires is 238 out of 337 collected questionnaires. The questionnaire utilization rate was 62%; 41% of men and 59% of women. The proportion of people aged 18–24 years is 66%, those aged 25–30 years is 22%, and those aged 31–35 years is 7%. Therefore, to some extent, this conclusion reflects the reality of young people's vaccination intention.

#### Reliability and Validity Analysis

To explain the internal consistency of the questionnaire, SPSS software was used to analyze the reliability and validity of the questionnaire. The basic composite reliability is calculated as:


(1) $$\begin{array}{r} \rho_{c}\;=\;\frac{{\left(\sum \lambda \right)}^{2}}{\left[{\left(\sum \lambda \right)}^{2}+\sum \left(\theta \right)\right]} \end{array}$$

In which λ is indicator loading, θ is indicator error variance.

As a typical index for composite reliability, Cronbach's coefficient is very important which is based on the standard coefficient.


(2) $$\begin{array}{r} \alpha \;=\;nr/\left[\left(n-1\right)r\;+\;1\right] \end{array}$$

In which *n* is the number of scale items in a questionnaire, and *r* is the average correlation coefficient between items, and α is the standard coefficient. Cronbach's coefficient can be expressed as:


(3) $$\begin{array}{r} Cronbach^{\prime }s\;\alpha \;=\;\frac{K}{K-1}\left(1-\frac{\sum_{i\;=\;1}^{K}\sigma_{Y_{i}}^{2}}{\sigma_{X}^{2}}\right) \end{array}$$

In which *K* is the number of scale items in a questionnaire, $\sigma_{X}^{2}$ is the variance of the total sample, and $\sigma_{Y_{i}}^{2}$ is the variance of the overserved sample.

In terms of reliability analysis, the Cronbach's coefficient of all variables in the questionnaire is 0.955, and the reliability coefficient value is >0.7, indicating that the questionnaire has relatively high reliability. Furthermore, it can be seen from Table 2 that, except for demand intensity variables, the Cronbach's coefficients of all variables are >0.7, indicating good reliability. The Cronbach's coefficient of demand intensity of 0.674 >0.65 can be explained by the minimum standard, which may be due to the continuous occurrence of COVID-19 when questionnaire data were collected. In general, the results of this questionnaire are reliable.

**Table 2:** Reliability analysis of each dimension.

**The variable name**	**Reliability statistics**	
	**Alpha**	**Number**
Professionalism	0.875	3
Morality	0.768	3
Interaction	0.844	4
Information content	0.876	7
Trust	0.895	6
Demand intensity	0.674	4
Perceived value	0.842	4
Vaccination intention	0.831	3

In terms of validity analysis, this study analyzed the content validity and structure validity, and KMO and Bartlett's test were adopted. KMO is calculated as:


(4) $$\begin{array}{r} KMO=BB/\left(AA+BB\right) \end{array}$$

where AA is the quadratic sum of the partial correlation coefficient between all the variables, and BB is the quadratic sum of the correlation coefficient between all the variables.

Moreover, Bartlett's can be calculated as:


(5) $$\begin{array}{r} \chi^{2}\;=\;-\;\left[n-\left(2p+11\right)/6\right]\ln|R|\;df\;=\;p\left(p-1\right)/2 \end{array}$$

where *n* is the number of records, *p* is the number of variables, and *R* is the correlation coefficient matrix.

In terms of content, this study draws on the classic and mature scale, conducts small-scale communication with relevant experts and medical professionals on relevant questions, adjusts and purifies some items of the scale, so the questionnaire meets the requirements in terms of content validity. In terms of structure, factor analysis was used to achieve this, and the gap between the KMO value of each variable and Bartlett's sphericity test value and relevant standards were compared by statistical software SPSS26.0 to ensure the structural validity of the fitting scale. As shown in Table 3, the KMO values of all the variables except Demand intensity and Morality were all >0.7, indicating that there were common factors among the variables. It was also significant (*P* < 0.005). The KMO values of Demand intensity and Morality were relatively low, which may be caused by the difficulty of perception of morality through the Internet except for occasional epidemic during questionnaire data collection.

**Table 3:** Variable KMO and Bartlett's sphericity test.

**Variable**	**KMO**	**Sig**
Demand intensity	0.674	0.000
Perceived value	0.841	0.000
Professionalism	0.722	0.000
Morality	0.696	0.000
Interaction	0.837	0.000
Information content	0.889	0.000
Trust	0.914	0.000
Vaccination intention	0.785	0.000

#### Hypothesis Verification of Independent Variables

In this study, structural equation models were used to verify the hypothesis, specifically using AMOS software. Structural equation analysis includes model fitting degree analysis and path analysis. The degree of fit analysis examines the degree of agreement between the hypothetical model and the model calculated from the sample data of the questionnaire. Path analysis examines correlation coefficients and significance levels between variables. Path analysis is based on path formulation, which can be expressed as:


(6) $$\begin{array}{r} y_{i}=\alpha +By_{i}+\Gamma x_{i}+\zeta_{i}\;var\left(\zeta_{i}\right)=\text{Ψ} \end{array}$$

In which, *y* are overserved endogenous variables, α are regression intercepts, *x* are overserved exogenous variables, ζ are disturbances, *B* and Γ are regression slopes, and Ψ are covariance matrix of disturbances.

In structural equation analysis, professionalism, morality interaction, information content, demand intensity, perceived value, and intention are incorporated into the complete model. The results show that some indexes are not in accord with absolute and relative fitting. Therefore, AMOS tested the path coefficient, the mediating effect, and the significant difference under standardization in the model, and found that among the seven path variable hypotheses based on the conceptual model, morality has no significant effect on trust, while professionalism and trust have relatively weak significance, as shown in Table 4.

**Table 4:** Test of significance of path coefficients of assumed model variables.

**Path**	**Standard coefficient**	**S.E**.	**C.R**.	***P***	**Significant difference**
Professionalism → Trust	0.192	0.062	0.332	0.017	A little Significant
Morality → Trust	−0.157	−0.284	−0.004	0.095	Non-significant
Interaction → Trust	0.413	0.192	0.590	0.001	Significant
Information Content → Trust	0.522	0.299	0.706	0.001	Significant
Demand Intensity → Perceived Value	0.826	0.664	0.925	0.001	Significant
Trust → Vaccination Intention	0.812	0.646	0.901	0.002	Significant
Perceived value → Vaccination Intention	0.299	0.154	0.453	0.001	Significant

The reason why H1 is not so strongly supported, maybe is that for professional medical Internet celebrities, their professional knowledge and fame are generally in the top list, it is like a default condition to ordinary people. Referring to the reason why H2 is not supported, maybe because these professional medical Internet celebrities are working in authoritative institutions, and their behavior and speech are always above a morality baseline, thus causing the weak relationship between morality and trust.

Based on the above analysis results, we modified the model. The model modification index (MI) value provided by AMOS can be used to optimize the fit degree of the model and improve the model. You can only modify one parameter at a time, adding or deleting paths to get the final optimized model. In this study, by eliminating the significance, this path Morality → Trust Vaccination Intention is insignificant in the hypothesis model of the test, and in the adjustment of the optimal model, it is found that removing that the path Professionalism → Trust Vaccination Intention, and adding the path Information Content Demand Intensity → Perceived Value Vaccination Intention, explanation ability of the model is improved obviously. The fitting indexes are shown in Table 5 after modification.

**Table 5:** Model fitting degree after modification.

**Indicators**	**Absolute fit**			**Degree of relative fit**		
	***X*^2^/*df***	**GFI**	**RMSEA**	**NFI**	**IFI**	**CFI**
Evaluation Good	<3	>0.9	<0.06	>0.9	>0.9	>0.9
Standard Fine	<3	>0.7	<0.08	>0.7	>0.7	>0.7
Correction model	2.210	0.715	0.072	0.712	0.821	0.821

As shown in Tables 6, 7, from the significance level and path coefficient between the revised variables, it can be seen that the model has been optimized, and the relationship between all variables presents a significant state.

**Table 6:** Corrected standard path coefficient and significance test.

**Path**	**Standard coefficient**	**S.E**.	**C.R**.	***P*-value**	**Significant difference**
Interaction → Trust	0.403	0.099	3.112	0.002	Significant
Information Content → Trust	0.385	0.096	3.034	0.002	Significant
Information Content → Demand Intensity	0.715	0.086	7.855	0.001	Significant
Demand Intensity → Perceived Value	0.910	0.121	8.639	0.003	Significant
Trust → Vaccination intention	0.645	0.138	6.110	0.002	Significant
Perceived Value → Vaccination Intention	0.361	0.066	5.114	0.001	Significant

**Table 7:** Modified full-path path coefficient and significance test.

**Standardized bootstrap mediation test**								
**Path**	**Effect of value**	**SE**	**Bias-corrected 95%CI**			**Percentile 95%CI**		
			**Lower**	**Upper**	***P***	**Lower**	**Upper**	***P***
StdIndA3	0.259	0.156	0.086	0.459	0.020	0.085	0.456	0.020
StdIndA4	0.245	−0.128	0.076	0.479	0.021	0.058	0.456	0.030
StdIndB1	0.327	0.325	0.196	0.474	0.001	0.180	0.459	0.001
StdIndB2	0.235	0.413	0.131	0.371	0.000	0.118	0.355	0.001

*IndA3, Interaction Trust Vaccinaon Intention; IndA4, Information Content Trusted Vaccination Intention; IntidB1, Demand Intensity Perceived Value Vaccination Intention; IndB2, Information Content Demand Intensity; Perceived Value Vaccination Intention*.

## Discussion

**Figure 2:**
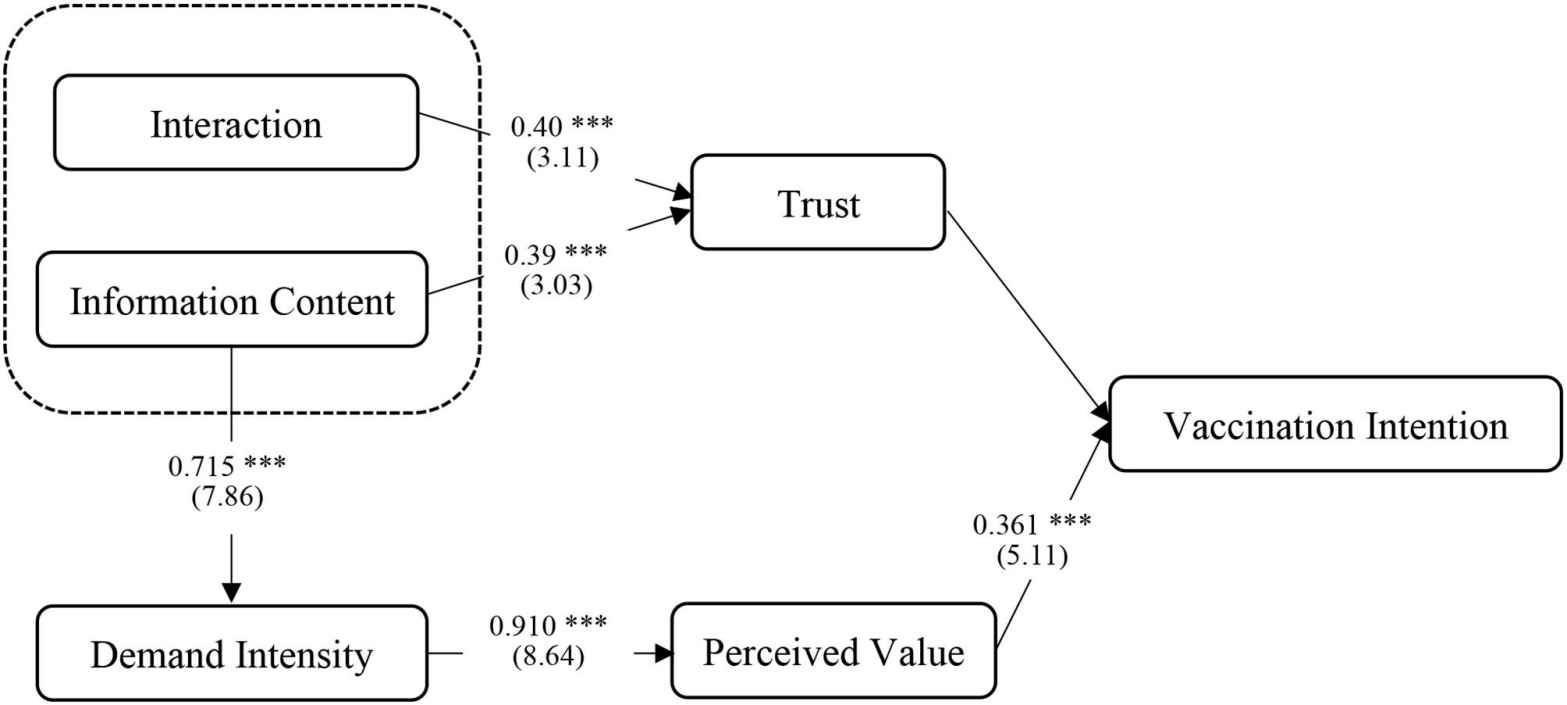
Modified structure model. ( ) Represents *t-*value; *** means *p* < 0.001.

According to the above empirical results, the modified structure model is displayed in Figure 2. According to the verification hypothesis, H1 and H2 were not proved, while H3, H4, H5, H6, and H7 were proved.

### The Importance of Characteristics of Internet Celebrity Medical Expert

In the model, the *t*-values of Professionalism, Morality, Interaction, and Information content on trust were 0.332, −0.004, 0.590, and 0.706, respectively. The characteristics of Information Content have the greatest influence on Trust, followed by Interaction, but the significance level is very significant (*P* < 0.005). The *t*-value of Morality was <0 and had no significant effect on Trust (*P* > 0.05). Professionalism had a significant effect on Trust (*P* = 0.017). To this end, in the revision of ideas, consider removing two independent variables Morality and Professionalism. At the same time, it is found that the optimization model can be improved by adding the path of Information Content → Demand intensity. After adjustment, the *t*-values of Interaction → Trust, Information content → Trust and Information content → Demand intensity were 3.112, 3.034, and 7.855, respectively, showing obvious significance (*P* < 0.005).

Acceptance analysis of hypothesis H3 and H4: Based on the above significance judgment, in this model, the hypothesis that the interaction of hypothesis H3 and the information content of hypothesis H4 have a significant impact on Trust is accepted, while the hypothesis that H2's morality has a significant impact on Trust is rejected. The reason may be: Interaction is the important embodiment of Internet celebrity features, the Internet celebrity medical expert. Interaction features can make people create more trust, this conforms to the social relationship advice from the theory, that is, for people to accept the effect of professional information and the impact of Internet celebrity medical expert, Internet celebrity medical expert should maintain interaction with the user and dwell into deeper emotions. The above conclusions also confirm that the network interaction found by Yuksel and Labrecque in the study is an important factor to strengthen the emotional connection between medical experts and the public, and the perception of the emotional relationship with medical experts can also be enhanced by improving the frequency and quality of social media interaction with medical experts (37).

At the same time, the path Information Content → Trust → Vaccination Intention has affirmed the importance of Internet celebrity medical experts on form and content of information.Internet celebrity medical experts with high-quality information and lively speech, and who are down-to-earth, strengthen emotional connection. The social public will rely on their subjective views to understand healthcare, and to determine its relationship with medical experts. This is consistent with the findings of similar studies (24). With good information content, people will even actively seek necessary and useful information from medical experts (38).

Analysis of rejection of hypotheses H1 and H2: Morality has not been proved, one of the reasons is that no normative scale has been concluded for reference in Morality. Second, unlike Professionalism, a person's morality can be proved officially. People are unable to perceive the morality of Internet celebrity medical experts. Different from previous research results, it is assumed that the characteristics of H1 specialization have a weak influence. The main reason may be social media and medical experts that perform the characteristics of Internet celebrities that weaken the role of professionalism. At the same time, medical terms are relatively specialized, and excessive professional terms used by medical experts when providing information on COVID-19 vaccine will make it difficult for ordinary people to understand, which may reduce people's information acceptance rate and thus affect their trust. In addition, professionalism characteristics may be more important to learning-oriented users.

### Influence Path of Vaccination Intention

The influence effects of the four paths influencing vaccination intention were all significant, and the most significant was the path through which demand intensity had an impact on vaccination intention through perceived value. IndA3, Interaction Dimension → Trust → Vaccination Intention; IndA4, Information Content → Trust → Vaccination Intention; IndB1, Demand Intensity → Perceived Value → Vaccination Intention; IndB2, Information Content → Demand Intensity → Perceived Value → Vaccination Intention. These four paths had effect value that was 0.259, 0.245, 0.327, and 0.235, respectively. IndA3 and IndA4 paths had weak significance (0.01 < *P* < 0.05), the indB1 and indB2 paths were significant (*P* < 0.005).

Acceptance analysis of hypothesis H5: The above analysis shows that the path of Demand Intensity → Perceived Value → Vaccination Intention has a greater impact on vaccination intention than the two paths of Interaction Dimension → Trust → Vaccination Intention and Information Content → Trust → Vaccination Intention. So, accept the hypothesis proposed by H5. Through research, Reichelt et al. also found that perceived benefits have a greater impact on Intention than other factors (39). In addition, the *t*-value of Demand Intensity compared to received value was as high as 8.65, significant at *P* < 0.00, which may be due to the frequent occurrence and long duration of the pandemic, leading to the increased importance of demand intensity for special products such as COVID-19 vaccine. Furthermore, it was found in the optimization model that the Information Content had a significant impact on the Demand Intensity (*P* < 0.005), and the *t*-value was 7.86, which could be understood as the effective information output of Internet celebrity medical experts, which significantly increased the public's demand for vaccines. The information content was an important aspect for Internet celebrities to perform their role.

This study found that Trust and Perceived value had significant effects on Intention. Among them, *t*-value of Trust → Vaccination Intention and Perceived Value → Vaccination Intention were 6.110 and 5.114, respectively, which had a significant influence on vaccination intention (*P* < 0.005). Therefore, we accept the hypothesis proposed by H6 and H7, and believe that trust and perceived value have significant positive effects on vaccination intention. However, different from the results of full-path analysis, the effect value of trust on vaccination intention (0.645) was almost 1.8 times that of perceived value (0.361). The reasons may be, on the one hand, that the measurement of the variable dimension of perceived value in this study is relatively simple, and other factors affect the perceived value; on the other hand, it may be that for the Novel Coronavirus vaccine, which is a brand-new object, the perceived value of the public is based on all the publicity rather than empirical data, which makes the trust in the Internet celebrity experts play a greater role.

## Conclusions

This study aimed to establish a scientific and effective model of the influencing factors that Internet celebrity medical experts have on the intention to vaccinate against COVID-19, to promote effective dissemination of professional information, alleviate “information epidemic,” and ultimately improve the vaccination rate of COVID-19 vaccine. Specifically, this study made an in-depth observation of the concept of Medical Internet celebrity from four different dimensions, studied the impact of the four dimensions on trust, and then proposed the introduction of synergy between demand intensity and perceived value in the context of COVID-19 normalization. Empirical research shows that an Internet celebrity medical expert has a significant role in COVID-19 vaccination through the way of promoting trust and perceived value, and information content and interaction have significant and positive effects on trust, and both have significant effects on vaccination intention. In the process of model modification, we found that the information content of Internet celebrity medical experts would eventually affect vaccination intention by influencing demand intensity and perceived value. In the context of the frequent occurrence of COVID-19, the influence of information content on demand intensity, and thus the way of influencing vaccination intention, should not be underestimated. Combined with the research conclusion, this study puts forward three suggestions.

First, Internet celebrity medical experts are needed. They are good for the COVID-19 vaccination, so to shape a few and provide resources to spread their influence is one of the important issues that government can do, and let everybody know that they are trustable and authorizable.

Second, information attractiveness is very important. Since the information content has a significant effect on trust and demand, we recommend that the information could be more interesting and closer to daily life, as well as create a temperature and lovely expert image, to achieve a more positive effect on people's intention on COVID-19 vaccination.

Third, creating vaccination demand is a key point. Based on the significant impact of information content on the demand intensity, we suggest grasping the timing of some local COVID-19 outbreaks affairs, and letting Internet celebrity medical experts to communicate information about infectivity and seriousness of the Virus to further enhance the intention to vaccinate against COVID-19.

## Data Availability Statement

The raw data supporting the conclusions of this article will be made available by the authors, without undue reservation.

## Author Contributions

JW and MZ: conceptualization, methodology, software, data curation, and writing-original draft preparation. FM and JC: data curation and writing reviewing and editing. JC and YX: writing-original draft preparation and funding. All authors contributed to the article and approved the submitted version.

## Funding

This research was funded by the National Social Science Fund of China, grant number 18BTQ050.

## Conflict of Interest

The authors declare that the research was conducted in the absence of any commercial or financial relationships that could be construed as a potential conflict of interest.

## Publisher's Note

All claims expressed in this article are solely those of the authors and do not necessarily represent those of their affiliated organizations, or those of the publisher, the editors and the reviewers. Any product that may be evaluated in this article, or claim that may be made by its manufacturer, is not guaranteed or endorsed by the publisher.
